# Histochemical Comparison of Human and Rat Lacrimal Glands: Implications for Bio-Engineering Studies

**DOI:** 10.1167/tvst.11.11.10

**Published:** 2022-11-14

**Authors:** John P. M. Wood, Glyn Chidlow, Luke A. Halliday, Robert J. Casson, Dinesh Selva, Michelle Sun

**Affiliations:** 1 Discipline of Ophthalmology & Visual Sciences, University of Adelaide, Adelaide South Australia, Australia; 2 South Australian Institute of Ophthalmology, Royal Adelaide Hospital, South Australia, Australia

**Keywords:** lacrimal glands (LGs), immunohistochemistry, histology, acinus, duct

## Abstract

**Purpose:**

The purpose of this study was to determine whether rodent lacrimal glands (LGs) represent a suitable surrogate for human tissue in bio-engineering research, we undertook a meticulous histological and histochemical comparison of these two tissues.

**Methods:**

Histological techniques and immunohistochemistry were used to compare the structure of adult human and rat LG tissues and the expression of key functional tissue elements.

**Results:**

Compared with humans, the rat LG is comprised of much more densely packed acini which are devoid of an obvious central lumen. Myoepithelial, fibroblasts, dendritic cells, T cells, and putative progenitor cells are present in both tissues. However, human LG is replete with epithelium expressing cytokeratins 8 and 18, whereas rat LG epithelium does not express cytokeratin 8. Furthermore, human LG expresses aquaporins (AQPs) 1, 3, and 5, whereas rat LG expresses AQPs 1, 4, and 5. Additionally, mast cells were identified in the rat but not the human LGs and large numbers of plasma cells were detected in the human LGs but only limited numbers were present in the rat LGs.

**Conclusions:**

The cellular composition of the human and rat LGs is similar, although there is a marked difference in the actual histo-architectural arrangement of the tissue. Further variances in the epithelial cytokeratin profile, in tissue expression of AQPs and in mast cell and plasma cell infiltration, may prove significant.

**Translational Relevance:**

The rat LG can serve as a useful surrogate for the human equivalent, but there exist specific tissue differences meaning that caution must be observed when translating results to patients.

## Introduction

The tear film serves a number of functions which are critical for the health of the ocular surface, including lubrication, cleansing, and provision of nutrients and other protective factors, as well as acting as a transparent, refractive medium to directly aid vision.^1^^–^^3^ A deficient tear film results in tear hyperosmolarity and insufficient ocular surface lubrication.^4^ The tear film is composed of three specific layers. The most abundant layer, by volume, is the middle aqueous layer which is secreted by the exocrine lacrimal gland (LG) and contains proteins, oxygen, and nutrients to protect the avascular cornea.^1^^,^^5^^,^^6^ Decreased LG function can be caused by a wide variety of factors, including aging,^7^ certain systemic or ocular medications,^8^^,^^9^ inflammatory conditions, such as dacryoadenitis or dacryocystitis,^10^ congenital,^11^ or acquired^12^ blockage of the nasolacrimal duct, tumor (head and neck) radiotherapy^13^ or autoimmune conditions, such as Sjögren's syndrome.^14^ The result of aberrant LG function is usually a reduced aqueous component of the tear film. This is termed aqueous-deficient dry eye,^15^^,^^16^ a specific form of dry eye disease which is a potentially debilitating condition^16^ and which is globally estimated to affect as much as 35% of the population.^17^^,^^18^ There are no adequate long-term treatments for aqueous-deficient dry eye disease except for application of tear substitutes, which generally fail to replicate the complex composition and properties of native tears.^19^

Due to the importance of the LGs in ocular function, this tissue has been the subject of anatomic study, both in physiological and pathological situations.^7^^,^^20^^–^^23^ The information provided by this body of work has allowed researchers to both understand basic mechanisms of LG action and to hypothesize that restoration of function, as a potential treatment option for dry eye syndrome, may be possible. Recently, for example, the LG has been cited as an ideal target for reparative bio-engineering.^24^^–^^28^ This would involve the reconstruction of this tissue using either bio-compatible implants seeded with cultured, native cells,^29^^–^^33^ or stem cell technologies.^34^^–^^37^ These technologies could theoretically restore the correct functioning of this tissue and therefore alleviate the complications that arise from its aberrant functioning.

Although the human LG has been rigorously studied, for ethical reasons and convenience, structure and function of this gland have also been investigated in other species, in particular, rodents and rabbits (see Schechter et al., 2010, for details).^21^ The study of non-human tissues to understand the human LG in both physiological and pathological situations has to necessarily make the assumption that there are negligible relevant inter-species differences. In the main situation, this is the case. The gross structural organization of the LG is similar across mammalian species with specialized epithelial cells making up acini that produce tear fluid and ducts which convey secretions to the ocular surface.^21^ Additionally, in humans, rabbits, and rodents, acini and ducts are surrounded by stromal tissue to provide structural and nutritive support.^21^ At a more detailed level, however, although human LG tissue does resemble that of the rabbit, as discussed in detail by Schechter and colleagues,^21^ it does vary significantly from the rodent equivalent, having acini which are less densely arranged, and with greater stromal mass. Further, in one of the only direct comparisons, tissue distribution of lectins was shown to differ in human LG versus the rat equivalent.^38^ These data suggest that although gross structure of the LG is similar between different mammalian species, specific differences can be observed.

Our laboratory is interested in the potential use of bio-engineering to rejuvenate pathological LG tissue. Specifically, we aim to extract cells from biopsy-sized samples of LG tissue collected from human patients and to propagate them with a view to repairing pathological tissue in situ.^25^^,^^29^^,^^30^ To provide a foundation for later studies on the human LG, however, we propose initially to use rodents as a suitable species surrogate. This would enable us to understand, in general, how LG cells respond to the experimental challenges that are involved in our procedures, before working with human tissue. In order to ascertain whether experimentation with rodent LG represents a valid proxy for human tissue, however, it is first necessary to carry out a detailed histochemical comparison of LG isolated from both species, to confirm that there are no significant differences which would complicate data extrapolation.

## Methods

### Human LG Tissue

Non-pathological orbital human LG tissue was routinely collected after being excised from patients either during a direct LG prolapse procedure or when there was evidence of LG prolapse during a blepharoplasty. None of the patients had a medical history of either LG disease or aqueous tear deficiency and there was therefore no documented history of LG dysfunction or pathology for any of the participants. At the time of collection, there was no evidence of LG inflammation. Collection and use of tissue was approved by the Central Adelaide Local Health Network Human Research Ethics Committee (HREC/13/RAH/445), and all participants provided written informed consent to the use of tissue for research purposes, after explanation of the nature and possible consequences of the study being undertaken. Tissue was collected into fresh, sterile Dulbecco's Modified Eagle Medium (DMEM; ThermoFisher Scientific Australia Pty Ltd, Scoresby, Victoria, Australia), at the time of excision, for transportation to the laboratory, and subsequently fixed by immersion for 24 hours in 10% (w/v) neutral-buffered formalin (NBF) before being processed for histological/immunohistochemical assessment. LG tissue was collected from five different donors for the assessments shown herein. Tissue was available from more elderly donors but was not included in order to avoid potential complications associated with aging. All donors were adult women; the ages of the donors were as follows: 40, 45, 47, 47, and 58 years. Only the LG of an 85 year old donor was noted to be fibrotic on dissection (but data from this donor was not included in this study).

### Animals

This study was approved by the Animal Ethics Committee of The University of Adelaide (Adelaide, South Australia, Australia) and conformed with the Australian Code of Practice for the Care and Use of Animals for Scientific Purposes, 2013, and with the ARVO Statement for the use of animals in vision and ophthalmic research.

Female adult Sprague-Dawley rats (6–9 months old) were bred by Laboratory Animal Services at the University of Adelaide, housed in a temperature- and humidity-controlled room with a 12-hour light/dark cycle, and were provided with food and water ad libitum. Rats were subjected to deep anesthesia by intraperitoneal injection of 100 mg/kg ketamine and 10 mg/kg xylazine, and were euthanized by transcardial perfusion with physiological saline, and, subsequently, with NBF while under anesthesia. After rats were humanely euthanized, the exorbital LGs were collected by careful dissection. Whole ocular globes with optic nerve attached, gastrointestinal (GI) tract, brain, livers and kidneys were also taken for the purpose of provision of positive tissues for immunohistochemistry. All tissues were immersion-fixed in NBF and transferred to 70% (v/v) ethanol until processing. Tissues were processed routinely before being embedded in paraffin wax and 5 µm thick sections were cut in each case for evaluation of tissue histologically and by immunohistochemistry. As with the human tissue, four individual rat LG samples were analyzed in the study.

### Histological Stains

Pieces of human and rat LGs were positioned as per their correct in situ tempero-nasal orientation, and transverse sections were prepared through the central region of the tissue. The gross tissue structure for both rat and human LGs was delineated by staining of deparaffinized and rehydrated sections using the Masson's trichrome procedure. This is similar in principle to a standard histological hematoxylin and eosin (H and E) stain in that it identifies cellular cytoplasm, nuclei, and extracellular matrix (ECM), except that it resolves the latter much more clearly from the background. To carry out this stain, initially, nuclei were stained with 0.5% (w/v) Celestin Blue/hematoxylin. This was followed with 1% (w/v) Biebrich Scarlet-1% (w/v) Acid Fuschin, in 1% (v/v) glacial acetic acid which stains all tissue components red. Subsequent differentiation with 5% (w/v) phosphotungstic acid removes the red color only from collagenous ECM components. The final step was treatment with 2.5% (w/v) Aniline Blue, with differentiation in 1% (v/v) acetic acid. This stains collagen fibers dark blue in color.

Mast cells were identified by staining of rehydrated sections with 0.1% (w/v) toluidine blue (pH 2.3) for 3 minutes. Toluidine blue stains mast cell granules red-purple (metachromatic staining) with the background tissue stained blue (orthochromatic staining).

### Mucins

Localization of acid mucins was achieved by routine staining of rehydrated sections with 1% (w/v) Alcian blue solution (pH 2.5) for 20 minutes. Alcian blue, at a pH of 2.5, stains all acid mucins deep blue, but does not react with neutral mucins. Localization of neutral mucins was achieved using the Periodic acid-Schiff (PAS) histological stain. In brief, rehydrated sections were treated with diastase for 20 minutes to digest any endogenous glycogen. Subsequently, sections were washed in running tap water, treated with 1% (w/v) periodic acid for 5 minutes, washed in distilled water, stained with Schiff's reagent for 10 minutes, washed in running tap water, and finally counterstained with hematoxylin. PAS stains all neutral mucins, plus acid mucins that contain significant quantities of sialic acid, a bright red magenta color.

### Immunohistochemistry

Colorimetric immunohistochemistry was per-formed as previously described.^39^^,^^40^ In brief, tissue sections were deparaffinized and treated with 0.5% (v/v) H_2_O_2_ to block endogenous peroxidase activity. Antigen retrieval was achieved by heating the sections in a microwave oven immersed in 10 mM citrate buffer (pH 6.0) for 10 minutes at 95 to 100°C. For localization of certain ECM proteins (laminin, collagen III, IV, and V), the Na-K-2Cl cotransporter (NKCC), and the tight junction markers ZO-1 and occludin-1, sections received an additional digestion for 15 minutes with proteinase K (20 µg/mL) to further unmask antigen sites. Subsequently, sections were incubated in primary antibody (see Table 1 for details), followed by consecutive incubations with biotinylated secondary antibody and streptavidin-peroxidase conjugate. Color development was achieved using 3,3′-diaminobenzidine and images of labeled sections were captured using a standard light microscope (BX51; Olympus, Mount Waverly, Victoria, Australia) with an attached vibration-free camera.

**Table 1.** Antibodies

Antibody	Host Species	Dilution	Company	Catalog # *Clone
ABCG2	Rat	1:3000	Santa Cruz Biotechnology	#sc-58224
α-SMA	Mouse	1:2000	Dako	M0851
Aquaporin-1	Rabbit	1:5000	Merck Millipore	#ab2219
Aquaporin-3	Rabbit	1:3000	Alomone Labs	#AQP-003
Aquaporin-4	Rabbit	1:3000	Santa Cruz Biotechnology	#sc-20812
Aquaporin-4	Rabbit	1:3000	Sigma	#HPA014784
Aquaporin-5 (human)	Mouse	1:500	Santa Cruz Biotechnology	sc-514022
Aquaporin-5 (rat)	Rabbit	1:3000	Alomone Labs	#AQP-005
CD3	Rabbit	1:2500	Dako	#A0452
CD31 (human)	Rabbit	1:1000	Spring Bioscience	*SP38
CD31 (rat)	Rat	1:500	Dianova	#DIA-310
CD34	Mouse	1:1000	Leica Biosystems	*NCL-L-END
CD79a	Mouse	1:1000	Dako	*HM57
CD138	Mouse	1:500	Dako	*MI15
claudin-5	Mouse	1:1000	Invitrogen	*4C3C2
Collagen III (human)	Rabbit	1:1000	Abcam	ab7778
Collagen III (rat)	Mouse	1:2000	Merck Millipore	AB3392
Collagen IV	Rabbit	1:1000	Abcam	ab6586
Collagen V	Goat	1:1000	Chemicon	ab781
Collagen VI	Rabbit	1:1000	Abcam	ab6588
Cytokeratin-8	Mouse	1:500	Dako	*35bH11
Cytokeratin-18	Mouse	1:1000	GeneTex	*C-04
eNOS	Mouse	1:1000	BD Transduction	#N30020
iba1	Goat	1:20,000	Novus Biologicals	NB100-1028
ki-67	Rabbit	1:750	Abcam	ab16667
Laminin	Rabbit	1:2500	EY Labs	AT 2404
MUC-4	Mouse	1:750	Santa Cruz Biotechnology	sc-33654
Nestin (human)	Mouse	1:1000	Merck Millipore	*10-C2
Nestin (rat)	Mouse	1:1000	BD Transduction	*rat-401
NKCC1/2	Mouse	1:10,000	DSHB	*T4
Occludin	Mouse	1:1000	Invitrogen	#33-1500
Pan-cytokeratin (1-8, 10, 14, 15, 16, 19)	Mouse	1:1000	Boehringer Mannheim	*AE1/AE3
Pan-cytokeratin (4-8, 10, 13, 14, 18, 19)	Mouse	1:750	ThermoFisher	*MA5
Pan-cytokeratin (4-6, 8, 10, 13, 18)	Mouse	1:1000	Cell Signaling Technology	*C-11
Synaptophysin	Rabbit	1:2000	Dako	#A0010
Tyrosine hydroxylase	Rabbit	1:2000	Merck Millipore	#ab152
Vimentin	Mouse	1:5000	Dako	#M0725
Vimentin	Rabbit	1:500	Cell Signaling Technology	#D21H3
ZO-1	Rabbit	1:1000	Invitrogen	#617300

For double-labeling fluorescent immunohistochemistry, visualization of one antigen was achieved using a three-step procedure (primary antibody, biotinylated secondary antibody, and streptavidin-conjugated AlexaFluor 488 or 594), whereas the second antigen was concurrently labeled by a two-step procedure (primary antibody, and secondary antibody conjugated to AlexaFluor 488 or 594). Sections were prepared as above, and then incubated overnight at room temperature in the appropriate combination of primary antibodies. On the following day, the sections were incubated with the appropriate biotinylated secondary antibody for the 3-step procedure plus the correct secondary antibody conjugated to AlexaFluor 488 or 594 for the 2-step procedure, followed by streptavidin-conjugated AlexaFluor 488 or 594. Sections were then mounted using anti-fade mounting medium and examined under standard microscope with epifluorescence optics (BX-61; Olympus) equipped with a scientific grade, cooled CCD camera.

Confirmation of the specificity of antibody labeling was judged by the morphology and distribution of the labeled cells, by the absence of signal when the primary antibody was replaced by isotype/serum controls, and by comparison with the expected staining antigen pattern in the LG and other tissues based on our own, and other, previously published results.

The relative abundance of immune cells in human and rat LG tissue was assessed by an independent observer in multiple sections and visually scored on a semiquantitative grading scale from not detectable (n. d.) to high prevalence (+++).

Cells that displayed positive labeling for Ki67 were counted in sections and data were compared between rats and humans using a non-paired Student's *t*-test (*n* = 12 per species).

## Results

### Tissue Structure

**Figure 1.**
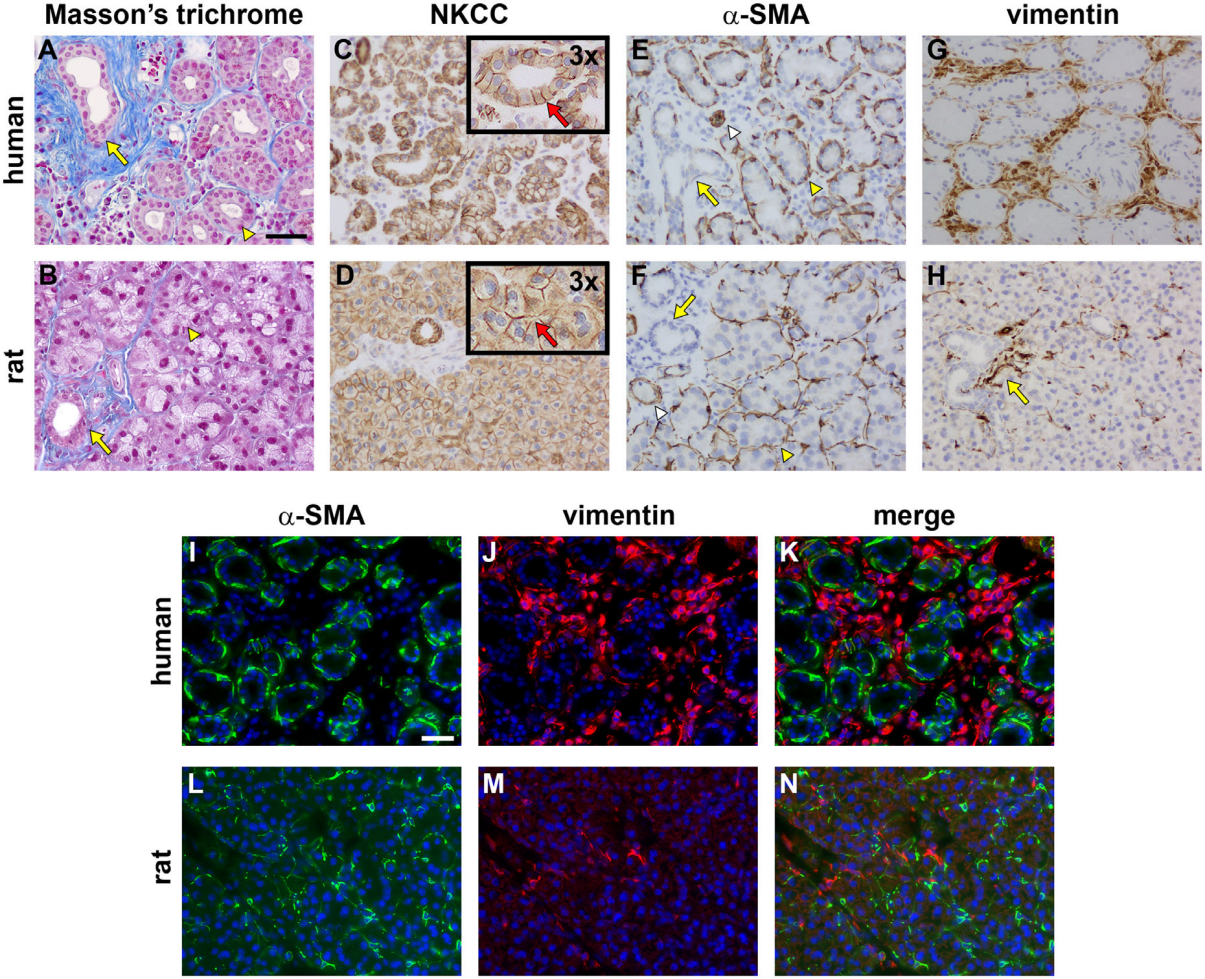
LG structure and main cell types in human and rat as revealed by Masson's trichrome staining and immunohistochemistry. Human LG (**A**) features loosely packed acini with sizeable lumina (*arrowhead*). Ducts (*arrow*), and to a lesser extent acini, are surrounded by connective tissue (*blue* elements). Rat LG (**B**) features acini that are tightly packed with barely discernible lumina (*arrowhead*). Connective tissue is concentrated around ducts (arrow). The Na-K-2Cl cotransporter (NKCC) is abundant on the epithelial cell basolateral membranes in acini and ducts in both human (**C**) and rat (**D**) LG (inset areas represent 3 times magnification of an area of an equivalently sized area of the original image; red arrows illustrate the different epithelial shapes). The α-smooth muscle actin (SMA)-labeled myoepithelial cells surround acini in human (**E**) and rat (**F**) tissues; some blood vessels are also labeled (*white arrowheads*). Vimentin-labeled fibroblasts within stromal tissue surrounding acini and ducts are numerous in human LG (**G**). In the rat, vimentin-positive fibroblasts tend to be concentrated around ducts (*arrow*), with few cells observed in acinar tissue (**H**). Double labeling immunofluorescence in human LG of α-SMA (**I**, *green*) with vimentin (**J**, *red*) reveals no discernible co-localization of these proteins (**K**, merge), indicating the absence of a significant population of myofibroblasts. Similar double-labeling immunofluorescence in the rat LG for α-SMA (**L**, *green*) and (**M**, *red*) also revealed no clear colocalization (**N**, merge). Scale bar: A to H = 60 µm; I to N = 60 µm.

Figure 1 shows an overview of the basic histo-architecture of both the human and rat LGs and identifies major structures and cellular components of these tissues.

Masson's trichrome staining reveals basic architecture of the tissues. Both human (see Fig. 1A) and rat (see Fig. 1B) LGs are composed of distinct clusters of associated cells, with interspersed collagen fibers (blue staining). Human tissue (see Fig. 1A) shows many distinct cellular bundles each surrounding a clear central lumen: these are accounted for by both acini and ducts. Rat tissue, however, is comprised of much more densely packed cell clusters mostly devoid of obvious lumina (see Fig. 1B). These clusters are the acini and where the few lumina are clearly visible, they indicate the presence of a duct.

Stromal collagen fibers are present throughout both human and rat glands, situated around each of the cellular bundles. These collagenous deposits have a much greater density when associated with a duct than an acinus (see Figs. 1A, 1B). The presence of increased collagen deposition, as localized by the Masson's trichrome stain, therefore, serves as a clear means to distinguish between the acini and ducts of the LG in a histological section.

NKCC was localized in both human and rat LG cells. The antibody used (see Table 1) recognized both NKCC1 and NKCC2. In the human LG, this transporter was localized to the epithelial cells surrounding each lumen, both in the acini and ducts (see Fig. 1C). In the much more densely packed rat LG tissue, NKCC was present in all putative epithelial cells in both acinar bundles and in ducts, not just those surrounding obvious central lumina (see Fig. 1D). NKCC labeling also demonstrated that the epithelia of the human acini and ducts, as well as the rat ducts, were cuboidal or columnar in nature, whereas those of the rat acini often appeared less columnar and more pyramidal in shape and were positioned closely together to limit the size of the central lumina (see Figs. 1C, 1D; see insets in each case for greater detail). In both human and rat LGs, where present, NKCC was located in basolateral membranes but not in apical or peri-luminal membranes.

Labeling of α-smooth muscle actin (α-SMA) revealed the presence of potentially contractile cells, such as myo-epithelia or myo-fibroblasts, in both human and rat LGs (see Figs. 1E, 1F) in identical patterns. Labeling for these cells surrounded epithelial cell clusters throughout the glands. In the main, α-SMA immunoreactivity was associated with acinar rather than ductal epithelial cells (see Figs. 1E, 1F). Some blood vessels were also labeled (see Figs. 1E, 1F; white arrowheads).

Immunolabeling for vimentin was undertaken to identify populations of fibroblasts, or other mesenchymal cells such as myo-fibroblasts. Labeling revealed vimentin to be associated with the stromal regions between epithelial cell clusters in both human and rat LG tissue (see Figs. 1G, 1H). In both tissues, there was a much greater quantity of vimentin immunoreactivity surrounding the ducts, rather than the acini. This accounts for the fact that there was a much greater density of labeling present in the human versus the rat LG, where, as described above, there was a relatively greater number of ducts present in relation to the acini.

To reveal more about the identity of the cells identified by both α-SMA- and vimentin-immunolabeling, double labeling was performed for both human (see Figs. 1I-K) and rat LG (see Figs. 1L, 1N), which revealed that each antigen was associated with a discrete cell population and did not colocalize.

### ECM Components

**Figure 2.**
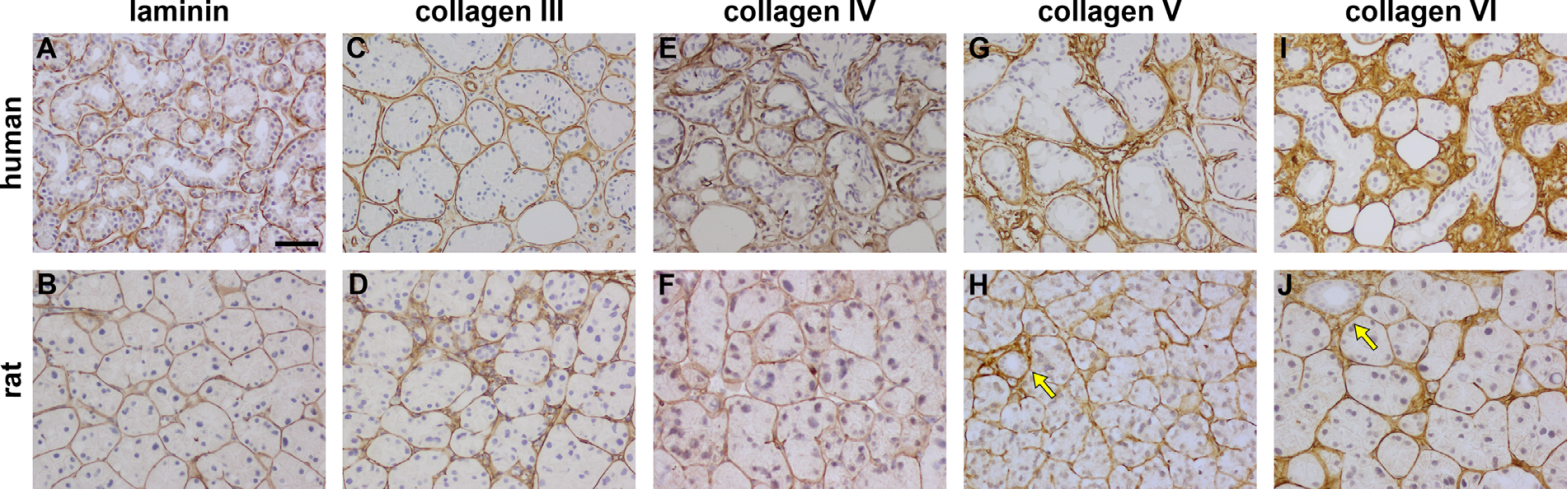
Representative photomicrographs the extracellular matrix proteins, laminin and collagen isoforms III to VI, in the human LG (**A, C, E, G, I**) and the rat LG (**B, D, F, H, J**). **A** and **B** Laminin is present in the epithelial basement membranes of acini and ducts. **C, D** Collagen III is clearly present and specifically associated with acinar and ductal epithelial basement membranes, as is collagen IV **E** and **F**, collagen V **G** and **H**, and collagen VI **I** and **J** which are also both present in larger amounts in the inter-acinar stroma (*arrows*). Scale bar = 60 µm.

Components of the stromal ECM in both human and rat LGs, including (pan-)laminin and different collagen isoforms (III-VI), were localized by immunohistochemistry (Fig. 2). Overall, there was a greater abundance of ECM in the human LG (see Fig. 2). This is consistent with the Masson's trichrome staining being of greater intensity around ducts rather than acini (see Figs. 1A, 1B) and with the fact that there are relatively less ducts per area in the rat tissue (see Figs. 1A, 1B). This is also consistent with the higher density of ECM-secreting fibroblasts within the larger stromal space in the human LG (see Fig. 1G) as compared with the rat LG (see Fig. 1H).

Human and rat LGs expressed laminin in very similar patterns, in the epithelial basement membranes of both acini and ducts (see Figs. 2A, 2B). In both cases, there is also a small amount of laminin present in the stroma, particularly around ducts, rather than acini.

**Figure 3.**
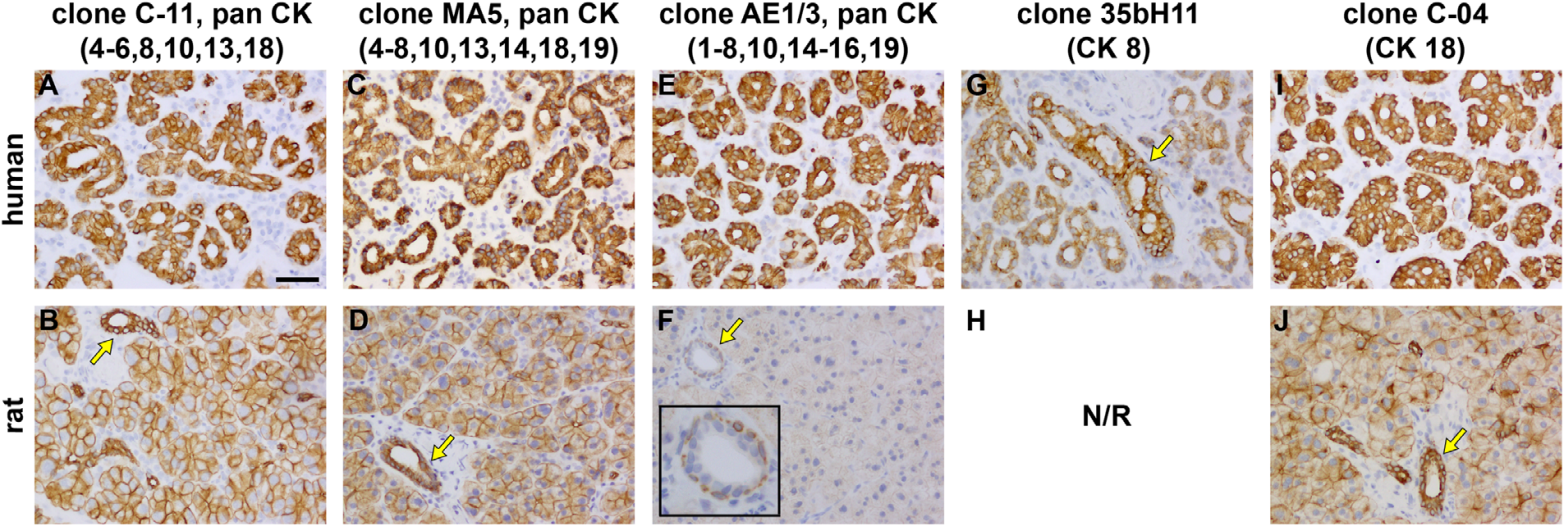
Representative photomicrographs of cytokeratin expression in the human and rat LGs. Clone C-11, a pan CK antibody that recognizes CKs 4-6, 8, 10, 13, and 18, robustly labels acini and ducts in human tissue (**A**). In the rat LG (**B**), clone C-11 likewise labels both acini and ducts, but the latter are delineated more intensely (*arrow*). Clone MA5, a pan CK antibody that recognizes CKs 4-8, 10, 13, 14, 18, and 19, is present in epithelial cells surrounding acini and ducts and displays an almost identical profile to clone C-11 in both human (**C**) and rat (**D**) tissue. Clone AE1/3, a pan CK that recognizes CKs 1-8, 10, 14-16, and 19, robustly labels both acini and ducts in human LG (**E**), but is absent from acini in rat tissue and only labels basal cells within ducts (**F**, *arrow* and *inset*). Clone 35bH11, which is specific to CK 8, robustly labels ducts (*arrow*) and variably labels acini in the human LG (**G**); however, the antibody is non-reactive in the rat (**H**), hence, no conclusions can be drawn. Clone C-04, which is specific to CK 18, displays very similar patterns of distribution to the pan CK clones C-11 and MA-5 in both the human (**I**) and rat (**J**) LGs. Scale bar = 60 µm.

Collagen III was clearly associated with epithelial basement membranes in both species (see Figs. 2C, 2D). In the rat LG, however, collagen III was also associated with the inter-acinar and inter-ductal stroma (Fig. 3D). Collagens IV, V, and VI were also associated with epithelial basement membranes, but all of these subtypes were also distributed throughout the stroma in both human and rat LGs (see Figs. 2E-J). There was a similar level of expression of collagens III and IV in human and rat glands (see Figs. 2C-F), but a relatively greater abundance of collagens V and VI in the former (see Figs. 2G-J). Comparison of the discernible expression level of each of the collagens (defined from most to least) in the human was collagen VI > V > IV > III, and in the rat was collagen VI > V = III > = IV.

### Cytokeratins

To compare the cytokeratin (CK) profiles of human and rat LG a panel of five different antibodies directed against CKs were used. All three “pan” CK antibody cocktails clones C-11, MA5, and AE1/3 were distributed almost identically in human LG (despite comprising slightly different complements of CKs; see Table 1), labeling epithelial cells robustly in both acini and ducts (see Figs. 3A, 3C, and 3E). In addition, in human tissue, the antibody specific to CK-18 (clone C-04; see Table 1) robustly labeled both acinar and ductal epithelia (see Fig. 3I). An antibody specific to CK-8 (clone 35bH11; see Fig. 3) showed positive reactivity in human LG acini and ducts, although the labeling was more intense in ducts and was not uniform in acini (see Fig. 3G).

In rat LGs, clones C-11 and MA5 (see Table 1 for details) yielded very similar results to the human with robust labeling of ducts together with moderate labeling of acini (see Figs. 3B, 3D). The third clone tested, clone AE1/3, yielded no specific labeling of acini, and only discrete labeling of basal epithelial cells in ducts (see Fig. 3F). The key difference between clones C-11 and MA5 compared with AE1/3 is that the latter clone does not recognize CK-18. These findings therefore strongly suggest that CK-18 is the principal cytokeratin expressed by rat acini. To examine this possibility further, an antibody specific to CK-18 (clone C-04; see Table 1) was also used: CK-18 yielded a pattern of immunolabeling analogous to the pan CK antibodies C-11 and MA5 (see Fig. 3J). Further confirmation that the rat LG tissue does not feature significant CK-8 expression was not possible as neither available clone specific to CK-8 were reactive in rat tissue (see Fig. 3, position H).

### Nervous Innervation

The nervous innervation of the human and rat LGs was investigated by immunolabeling for the presence of both synaptophysin, an integral membrane protein localized within the presynaptic vesicles of all nerves, and, tyrosine hydroxylase (TH), which specifically demarcates adrenergic nerves. For positional clarity, both antibodies were co-labeled with α-SMA, which demarcated putative myo-epithelial cells (see Fig. 1).

**Figure 4.**
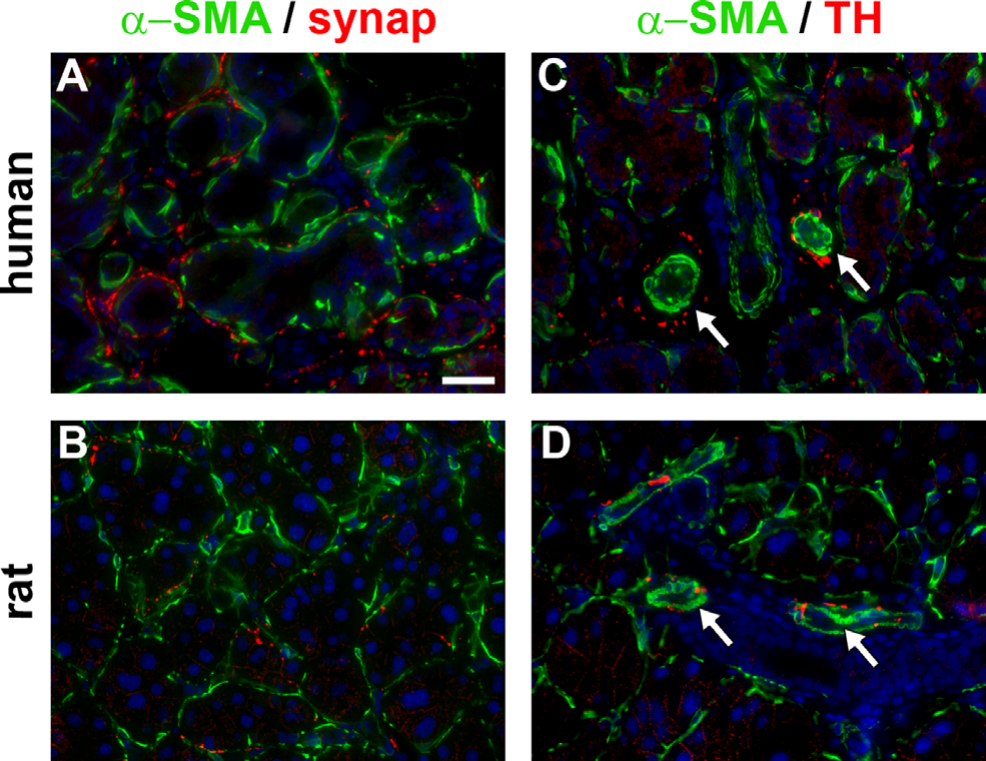
Innervation of the human and rat LGs. Double labeling immunofluorescence of α-smooth muscle actin (SMA, *green*) with synaptophysin (synap, *red*) in the human (**A**) and rat (**B**) LGs. Synaptophysin-positive fibers are present within stromal tissue and surround many acini where they lie predominantly in close proximity to α-SMA-labeled myoepithelial cells and blood vessels. Double labeling immunofluorescence of α-SMA (*green*) with tyrosine hydroxylase (TH, *red*) in the human (**C**) and the rat (**D**) LGs. TH-positive sympathetic fibers are relatively sparse in distribution and are predominantly found adjacent to blood vessels (*arrows*) plus a small number of acini in both the human and rat LGs. Scale bar = 60 µm.

In both the human and rat LGs, synaptophysin immunoreactivity localized predominantly surrounding acini, in close proximity to myoepithelial cells and some blood vessels (Figs. 4A, 4B). TH immunoreactivity was sparse in both human and rat LGs, being essentially restricted to blood vessels and surrounding a small number of acini (Figs. 4C, 4D).

### Immune Cells

**Figure 5.**
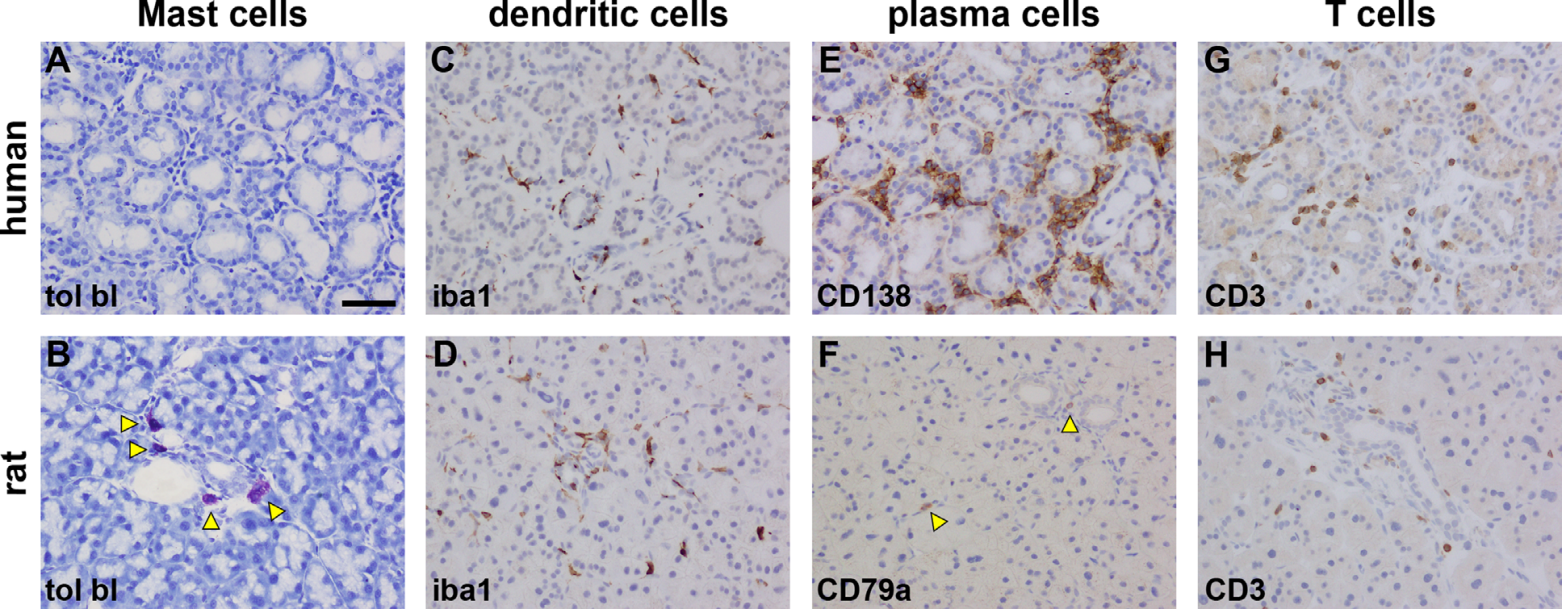
Representative photomicrographs displaying detected immune cells in the human and rat LGs, as revealed by toluidine blue (tol bl) staining and immunohistochemistry. No Mast cells were evident in human LGs (**A**). Rat lacrimal featured occasional Mast cells (*arrowheads*) that were most commonly observed around ducts. Dendritic cells, labeled by iba1, were numerous in both human (**C**) and rat (**D**) LGs. Plasma cells, labeled by CD138, were abundant in stromal spaces in human LG (**E**). In contrast, in the rat LGs, plasma cells, labeled by CD79a, were scarce (see *arrowheads*). T cells, labeled by the pan T cell marker CD3, were moderately plentiful throughout human LG (**G**), but were less widespread in the rat LG (**H**), being concentrated around ducts. Scale bar = 60 µm.

Mast cells were identified by toluidine blue staining. No mast cells were detectable in human LG (Fig. 5A, Table 2); however, a small number of these cells were localized to the peri-ductal stroma in the rat (see Fig. 5B, Table 2).

**Table 2.** Summary of Relative Proportions of Immune Cells in Human and Rat LGs

	Mast Cells	Dendritic Cells	Plasma Cells	T Cells
Human	n.d.	++	+++	++
Rat	+	++	+	+

n.d., not detected.

Dendritic cells, plasma cells, and T-cells were identified by immunohistochemistry. Dendritic cells were labeled with an antibody to iba1, a highly conserved immunomodulatory protein also expressed by all other cells of the monocyte/macrophage lineage.^41^ Iba1-immunolabeling was detected in very similar patterns in numerous cells in both human and rat LGs, predominantly located adjacent to the luminal epithelia of ducts (see Figs. 5C, 5D, Table 2). Iba1-immunoreactive cells did not feature the morphology of monocytes/macrophages and were therefore likely to exclusively be dendritic cells.

CD138 (syndecan-1) is a classical marker of differentiated plasma cells.^42^ Assessment of CD138 immunoreactivity in human LG identified a sizeable population of plasma cells in the stroma, adjacent to the outer face of the basement membranes of the ductal, but not acinar, epithelia (see Fig. 5E, Table 2). Neither antibody to CD138 were reactive in rat tissues, as evidenced previously by testing in the spleen, which is replete with plasma cells.^43^ Therefore, a different antibody was used for plasma cell identification in rat LG, namely CD79a, which recognizes all B cells, of which plasma cells form a subpopulation.^44^ Large numbers of CD79a-positive cells were detected in rat spleen and the GI tract (Supplementary Fig. S1), verifying the utility of this antibody in rat tissues. In rat LGs, only a small number of plasma cells were identified relative to human LGs (see Fig. 5F, Table 2). As with the human LGs, these cells were present in the peri-ductal stroma, adjacent to the epithelial basement membrane (see Fig. 5F).

CD3 is a pan T-cell marker.^45^ Assessment of CD3 immunoreactivity in both human and rat LG (see Figs. 5G, 5H, Table 2) sections revealed similar patterns of distribution, with a limited population of T cells present in the peri-ductal stroma, adjacent to the basolateral surfaces of the epithelia. The presence of a greater number of detectable cells in the human LG likely reflects the great ratio of ducts to acini, as described above.

### Aquaporins

The aquaporins (AQPs) are a family of integral membrane proteins that conduct water across plasma membranes and which play a crucial role in the maintenance of secretory exocrine gland function.^46^ The most studied AQPs in exocrine glands, for which there exist validated antibodies, are AQPs 1, 3, 4, and 5; these were the isoforms examined here.

**Figure 6.**
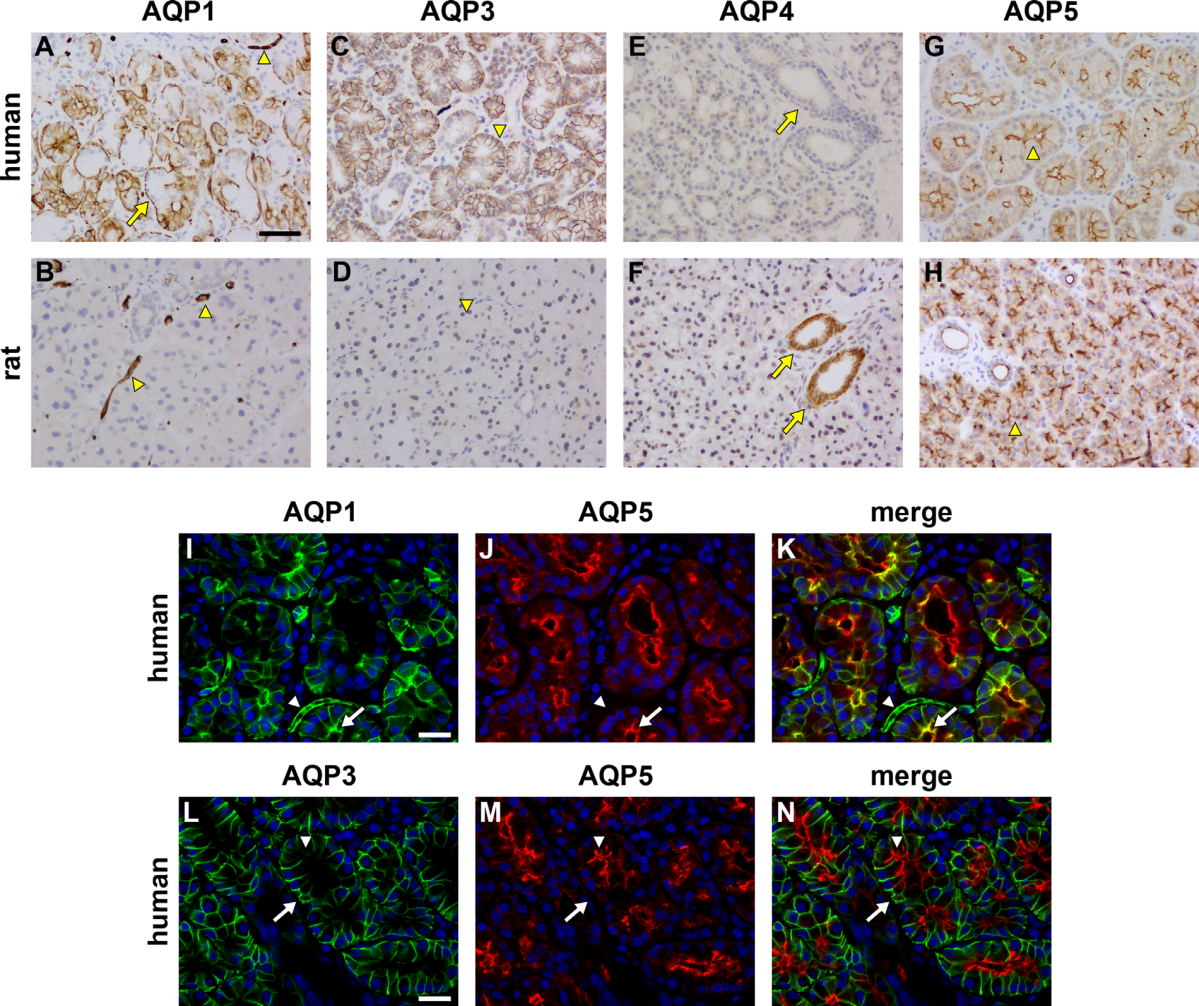
Representative photomicrographs of aquaporin (AQP) expression in human and rat LG. AQP1 expression in human LG is widespread, localizing to myoepithelial cells surrounding acini (*arrow*), blood vessel endothelia (*arrowhead*), and inconsistently by acini (**A**). In the rat LG, AQP1 expression is restricted to the endothelia of blood vessels (**B**). AQP3 localizes to the baso-lateral membranes of acini (*arrowhead*) in the human LG (**C**), but is absent from rat tissue (**D**). AQP4 expression was not observed in acini or ducts (*arrow*) in the human LG (**E**), but is robustly expressed within ducts of rat tissue (**F**, *arrows*). In both the human (**G**) and rat (**H**) LGs, AQP5 localizes to the apical surface of acini (*arrowheads*) and ducts. Double labeling immunofluorescence of AQP1 (**I**, *green*) with AQP5 (**J**, *red*) reveals colocalization of these proteins within some apical membranes of acini (*arrow*), but no colocalization in blood vessels (**K**, merge, *arrowhead*). Double labeling immunofluorescence of AQP3 (**L**, *green*) with AQP5 (**M**, *red*) reveals no discernible colocalization of these proteins (**N**, merge, *arrow*, *arrowhead*). Scale bar: **A** to **H** = 60 µm; **I** to **K** and **L** to **N** = 20 µm.

AQP1 was localized in cells throughout the human LG (Fig. 6A), in particular, the basolateral and apical surfaces of epithelia in most, but not all, acini and ducts, blood endothelia, and the myoepithelial cells surrounding acini. Interestingly, expression of AQP1 was markedly different in the rat LG, where labeling was confined to vascular endothelial cells (Fig. 6B). The validity of the AQP1 antibody in human and rat tissues was confirmed using appropriate positive control tissues (Supplementary Figs. S2A-C).^47^

AQP3 localized to the basolateral surfaces of acinar, but not ductal, epithelial cells throughout the human LG (Fig. 6C); however, this isoform was completely absent from rat LG (Fig. 6D). The validity of the AQP3 antibody in the rat was confirmed using the GI tract, which displayed expression of AQ3 in the basolateral membrane of surface epithelial cells (Supplementary Fig. S2D), as previously reported.^47^

AQP4 immunoreactivity was absent from human LG (Fig. 6E), but robustly labeled the basolateral surfaces of ductal, but not acinar, epithelial cells in the rat (Fig. 6F). Positive control labeling in human retina and brain, as well as rat retina (Supplementary Figs. S2E-G) demonstrated reactivity of the antibody used in both species.

AQP5 immunoreactivity was of an identical nature in human and rat LGs, being detected specifically in apical membranes of both ductal and acinar epithelial cells (Figs. 6G, 6H).

Immunohistochemical double-labeling was undertaken in human LG sections to confirm the epithelial compartmentation of AQP immunoreactivities. Figures 6I to 6K, shows co-labeling of AQP1 and AQP5. There is some colocalization of AQP1 and AQP5 in the apical surfaces of epithelial cells, although in most situations, AQP1 and AQP5 have discrete intracellular locations (Fig. 6K). Figures 6L to 6N show double-labeling of AQP3 and AQP5. As expected, there is no colocalization between AQP3 and AQP5, with the former restricted to the basolateral surface and the latter to the apical surface of epithelial cells (see Fig. 6N).

### Tight Junctions

Tight junctions are composed of a multi-protein junctional complex, creating a seal between adjacent cells to prevent paracellular leakage of fluids and solutes. Two of the major trans-membrane constituents of tight junctions are the claudin multi-protein family and occludin, and these associate with intracellular peripheral anchoring proteins such as the zonula occludens (ZO) proteins. To assess the presence of tight junctions in human and rat LGs, we examined localization of three key constituent proteins: ZO-1, occludin, and claudin-5.

**Figure 7.**
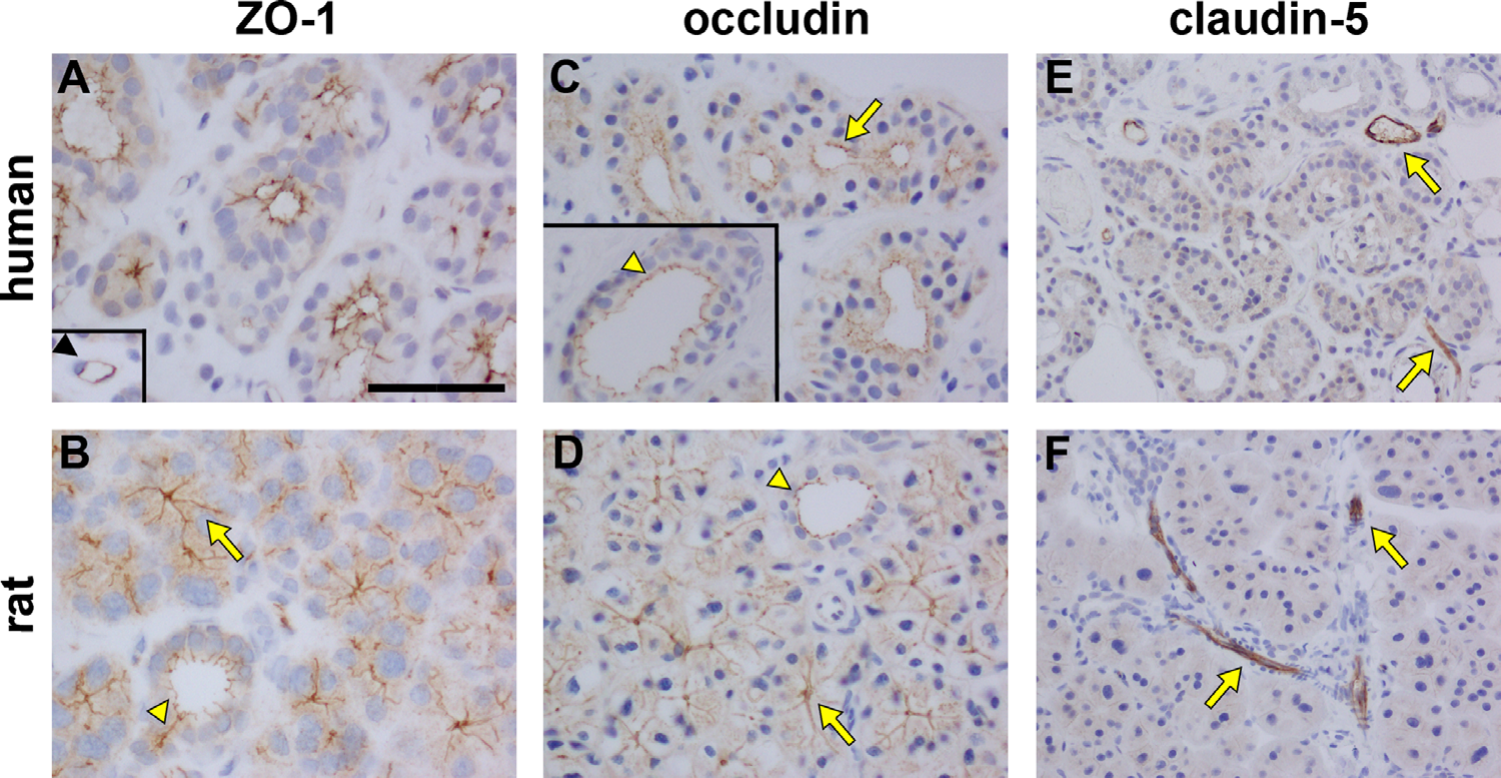
Representative photomicrographs of tight junction proteins in human and rat lacrimal gland. In both human and rat LG, ZO-1 (**A, B**) and occludin (**C, D**) both localized to the apical surface of acini (*arrows*) and ducts (*arrowheads*), as well as to vascular endothelium (*black arrowhead*). Claudin-5 was not expressed in acini or ducts in either the human (**E**) or rat (**F**) LGs with expression being restricted to the vascular endothelium (*arrows*). Scale bar = 30 µm.

ZO-1 was present on the apical faces of ductal and acinar epithelial cells in human and rat LG (Figs. 7A, 7B). In both cases, no labeling was detected on the basal surface of the epithelia. Labeling for occludin was identical to that of ZO-1 in both species (Figs. 7C, 7D). As described for Figure 1, there are obvious central lumen in human LG acini, but these are lacking or are extremely narrow in the rat, where the apical surfaces are convoluted and invaginate almost to the basement membrane. Thus, the apical surface labeling of both ZO-1 and occludin in the epithelial cells of the rat acini appeared to extend along their lateral edges to their base (see Figs. 7B, 7D). In the case of the rat ductal epithelia, the apical labeling was much more distinct, because a clear central lumen was observable.

Immunohistochemical labeling of claudin-5 revealed a localization pattern which was distinct from ZO-1 and occludin. In both human and rat LG sections, claudin-5 was restricted to vascular endothelial cells (Figs. 7E, 7F), and not to acinar/ductal epithelia.

### Blood Vessels

**Figure 8.**
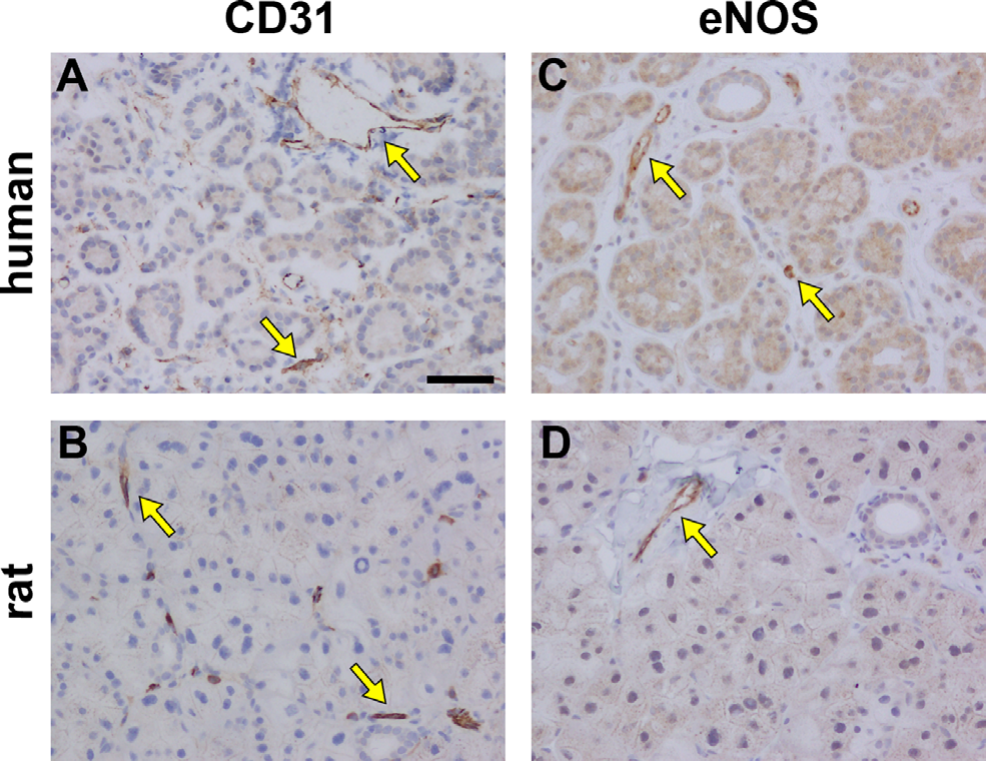
Representative photomicrographs of blood vessels in the human and rat LG. Blood vessels in the human (**A**) and rat (**B**) LGs were demarcated by expression of CD31 (*arrows*). Endothelial nitric oxide synthase (eNOS) was expressed by the vascular endothelium in both the human (**C**) and rat (**D**) LGs (*arrows*). Scale bar = 60 µm.

To demarcate blood vessels, vascular endothelial cells were labeled immunohistochemically for both CD31 and for the endothelial isoform of nitric oxide synthase (eNOS). In both human and rat LGs, each of CD31 (Figs. 8A, 8B) and eNOS (Figs. 8C, 8D) specifically labeled structures resembling blood vessels. There was no obvious difference in abundance in either marker between rat and human LGs.

### Mucins

**Figure 9.**
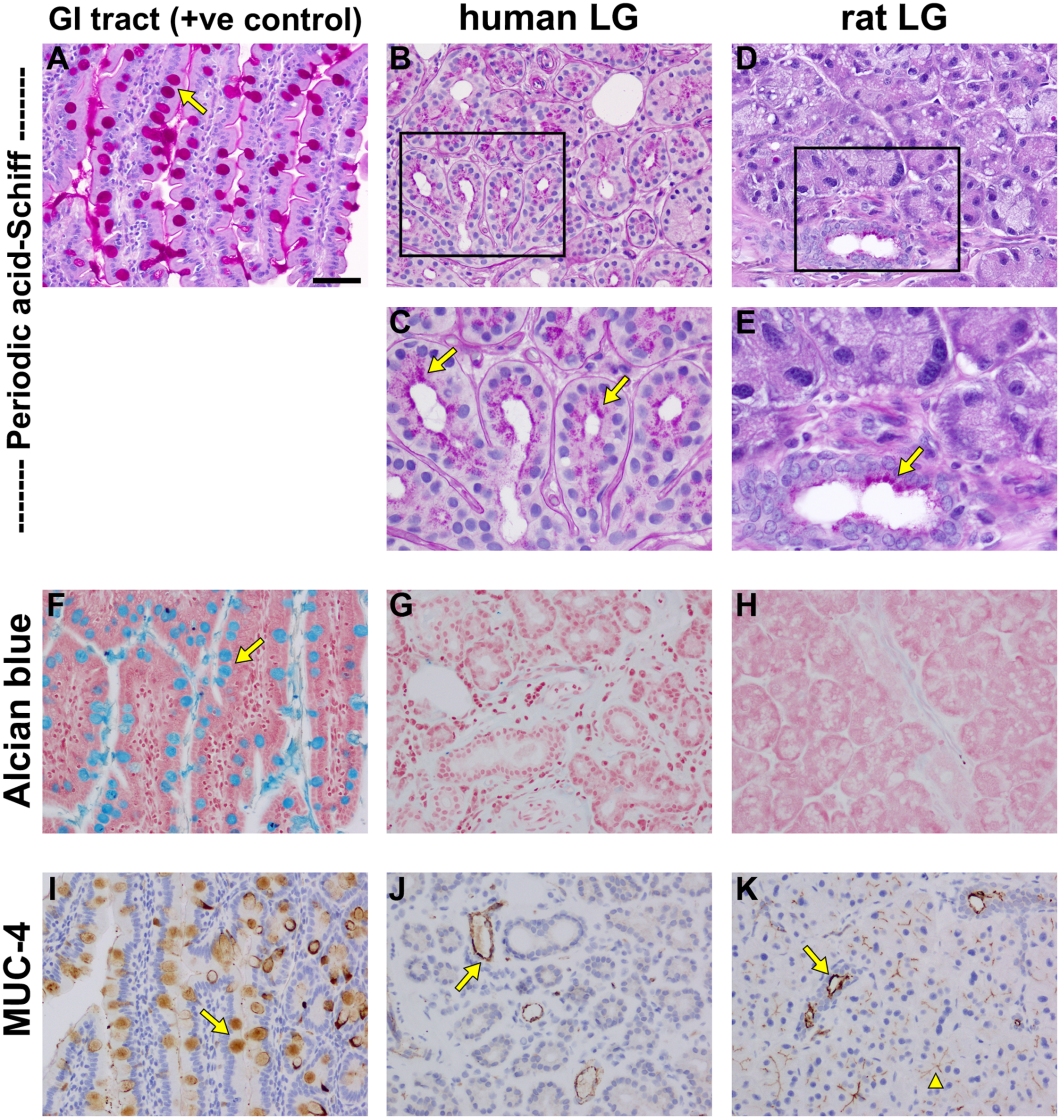
Representative photomicrographs of mucins in the human and rat LG, as revealed by histological stains and immunohistochemistry. Periodic acid-Schiff stains neutral mucins bright magenta, as shown by staining of goblet cells (*arrow*) in the positive control tissue, in the gastrointestinal tract (**A**). In human LG (**B**, and **C** higher magnification inset), positive staining is observed in the apical region, extending into the lumen, in the majority of acini (*arrows*) and ducts. In rat LGs (**D**, and **E** higher magnification inset), positive staining is observed only in ducts (*arrow*), not acini. Alcian blue, pH 2.5, stains acid mucins blue, as shown by staining of goblet cells (*arrow*) in the positive control tissue, in the gastrointestinal tract (**F**). In neither human (**G**) nor rat (**H**) LGs, was there any unambiguously positive staining for Alcian blue. MUC-4 immunolabeling is present in goblet cells (*arrow*) in the positive control tissue, in the gastrointestinal tract (**I**). In the human LG (**J**), MUC-4 labeling is observed only in ducts (*arrow*). In the rat LG (**K**), MUC-4 labeling is likewise evident in ducts (*arrow*), but is also present at the apical surfaces of the majority of acini (*arrowhead*). Scale bar: **A**, **B**, **D**, **F** to **K** = 60 µm. **C**, **E** = 30 µm.

The PAS histological stain was used to demarcate the presence of neutral mucins in LG tissues. To validate and optimize the PAS procedure, sections of the GI tract were initially stained; the correct identification of neutral mucins in goblet cells lining the tract confirmed the utility of this method (Fig. 9A). In the human LG, positive labeling for neutral mucins was identified in the majority of acinar and ductal epithelial cells. This intracellular staining was present in the apical portion of the epithelial cells and extended up to the luminal cell surface (Figs. 9B, 9C). In the rat LG, only ductal and not the acinar epithelial cells were stained for the presence of neutral mucins, with labeling again detectable in the apical portion of epithelial cells, extending to the luminal face (Figs. 9D, 9E).

Alcian blue stain was used to demarcate acid mucins, first in the GI tract, where it specifically identified a population of goblet cells (Fig. 9F), and then in the LG. There was no unambiguous positive alcian blue staining in either the human or rat LGs (Figs. 9G, 9H), indicating minimal acid mucin production in these tissues.

Finally, we examined the expression of a key individual mucin: the prominent epithelial membrane-bound isoform, mucin-4 (MUC-4). Positive identification of this mucin was again demonstrated in goblet cells in the GI tract (Fig. 9I). In the human LG, MUC-4 was observed in ductal epithelial cells (Fig. 9J). In the rat LG, MUC-4 was present at the apical surfaces of both ductal and acinar epithelia (Fig. 9K).

### Progenitor Cells

Investigation into the presence of progenitor or stem cells in exocrine glandular tissue is an ongoing research area because of the clinical possibilities that this may engender. Mouse^48^ and human^49^ LGs have been shown to harbor cells expressing progenitor cell markers. The present study investigated the potential presence of progenitor cells by examining expression of four proteins which are associated with these cells.

**Figure 10.**
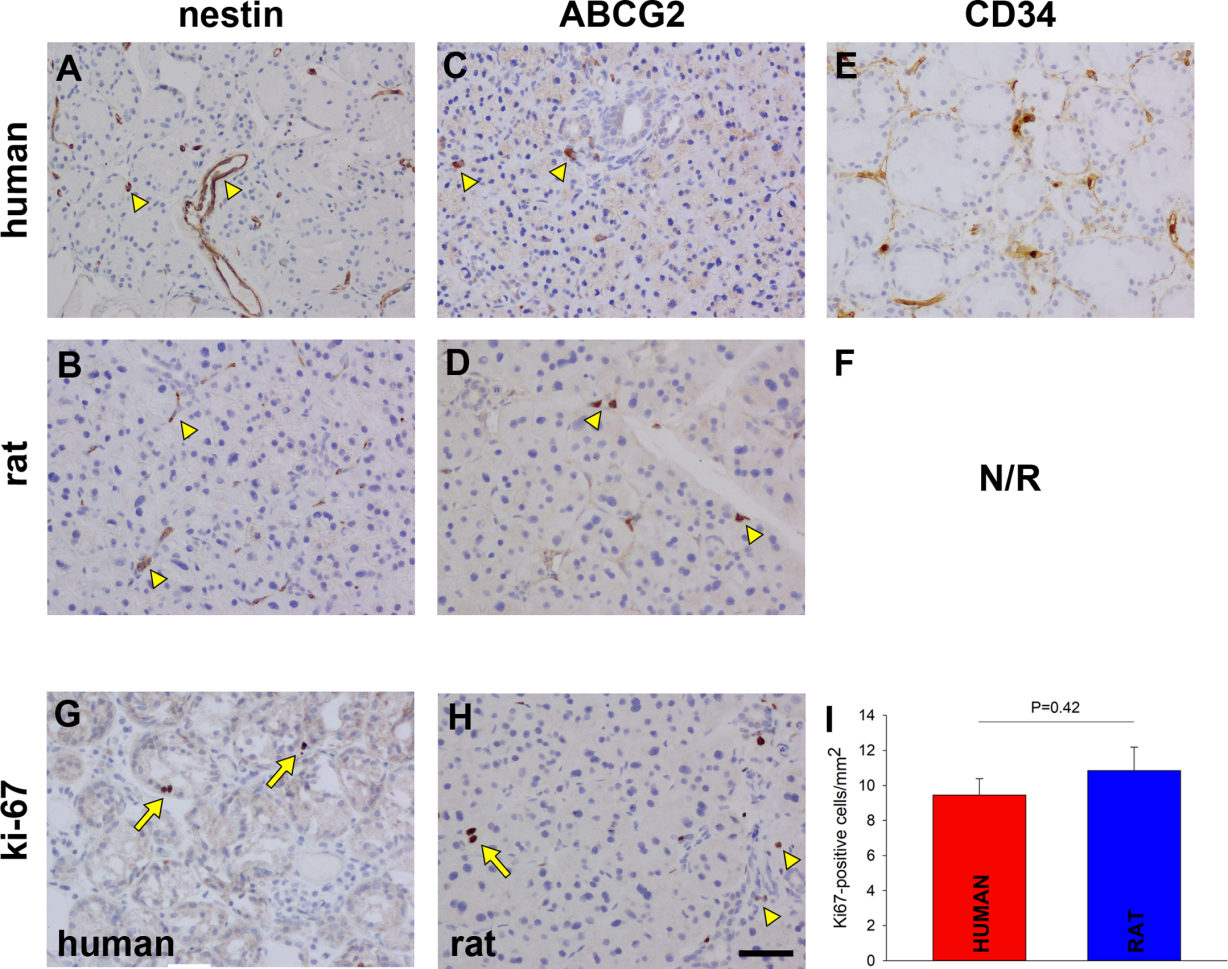
Representative photomicrographs of putative progenitor cell markers and associated proteins in the human and rat LGs. Expression of nestin appeared to be restricted to blood vessels (*arrowheads*) in both the human (**A**) and rat (**B**) LGs. Occasional ABCG2-positive cells were present within both the human (**C**) and rat (**D**) LGs (*arrowheads*). In the human LG, expression of CD34 was observed in blood vessels plus other cells surrounding acini (**E**). CD34 was non-reactive in rat tissue (**F**). Ki-67-positive cells were infrequently encountered in both the human (**G**) and rat (**H**) LGs, being typically located within acini (*arrows*) when observed. Smaller ki-67 positive cells were evident in rat LG in close proximity to ducts (*arrowheads*). Comparison of numbers of Ki-67-positive cells in the human and rat LGs revealed that there was no statistically significant difference between the two species (non-paired Student's *t*-test; *n* = 12 for each; **I**). Scale bar = 60 µm.

Nestin is an intermediate filament protein expressed during development or by glial cells or multi-lineage progenitor cells in the nervous system, including the eye.^50^ Vascular endothelial cells can also express this protein.^51^ Positive control labeling for nestin was confirmed in astrocytes in rat optic nerve and human optic nerve head (Supplementary Figs. S3D, S3E). In both human and rat LGs, nestin immunoreactivity was restricted to structures that had the histological appearance of blood vessels (Figs. 10A, 10B). The distribution of nestin immunoreactivity was analogous to that of CD31- and eNOS-positive blood vessels (see Fig. 8).

ATP-binding cassette super-family member 2 (ABCG2) is an ATP-binding cassette transporter that removes a wide variety of endogenous and exogenous substances out of cells,^52^ particularly in the kidneys or the GI tract. ABCG2 is also often expressed in stem cell populations,^53^ such as human limbal epithelial cells.^54^ The validity of the ABCG2 antibody in the present study was confirmed by positive immunohistochemical labeling of ABCG2 in the proximal tubules of the kidneys, epithelial apical membranes in the GI tract, and hepatic bile canalicular membranes (Supplementary Figs. S3A, S3C). In both human and rat LG sections, ABCG2-immunoreactivity was detected in an occasional population of cells in the stromal regions between acini (Figs. 10C, 10D).

CD34 is expressed by vascular endothelial cells, hemopoietic stem/progenitor cells, and some fibroblasts.^55^ Furthermore, the CD34-positive fibroblast in many organs is thought to represent an uncommitted cell capable of multidirectional mesenchymal differentiation.^55^ In the current study, CD34-immunoreactivity was detected in blood vessels and some fibroblast-like cells in the stromal tissue surrounding acini (Fig. 10E). CD34 was non-reactive in rat tissue (Fig. 10F).

Ki67 is a protein predominantly associated with proliferating cells; in a non-dividing tissue, this can denote the presence of either stem or progenitor cells. A small number of Ki67 immunoreactive cells were observed in both human and rat LGs (Figs. 10G, 10H). Where present, these were invariably associated with the acinar epithelium, in the human (see Fig. 10G), but both acinar and ductal epithelium, in the rat (see Fig. 10H). Analysis of the numbers of Ki67-positive cells in rat and human LG sections revealed that there was no significant difference between them (*P* = 0.42, by non-paired Student's *t*-test; *n* = 12 for each; Fig. 10I).

## Discussion

### Structure of the LG

In terms of the tissue architecture, human and rat LGs are similar. Both consist of a compound tubulo-acinar arrangement, comprising clusters of individual acini which act as the secretory units, linked via a series of ducts. We observed that visible ducts were present that had lumina of varying size, in both human and rat LGs. However, we did not define whether ducts were intralobular, interlobular, intralobar, or interlobar, and therefore referred to all of these structures generically as ducts. Identical patterns of labeling for the endothelial marker proteins, eNOS and CD31 and the endothelium-specific tight junction protein claudin-5, were detected in the human and rat LGs, demonstrating that both tissues were equally well vascularized.

It has previously been documented that subtle differences in the histo-architecture of human and rat LGs exist.^21^ LG differences found in the present study corroborate these findings: the human LG is composed of more loosely packed acinar cell clusters than the rat^56^^–^^58^ and has a relatively larger proportion of ducts per unit area. One obvious difference was the lack of a clear central lumen in the rat acinar unit. Although acinar lumina were not clearly visible, they are present. In fact, rat acinar epithelia are tightly packed along their lateral faces, leaving little room for a central lumen. We demonstrated this by showing that acinar tight junctions, as visualized by occludin- and ZO-1-labeling, were clearly observed in acini (and ducts), located along epithelial margins right into the center of these cell clusters. Previous work has documented that rodent LG acinar epithelium is composed of tightly packed pyramidal cells,^58^ as compared to the human cuboidal/columnar type.^21^^,^^23^^,^^59^ With a very narrow lumen in the rat LG acini, it would be expected that the apical face of the epithelia would have limited length, and, therefore, a reduced surface area available for secretion. This is, however, not the case, because these cells have been shown by electron microscopy to possess highly convoluted apical surfaces, often invaginating from the lumen to close to the basement membrane.^21^ This allows a large surface area for secretion, even though the associated lumen appears narrow. The structural characteristics of the rat LG, therefore, do allow for recapitulation of human LG function.

The distribution of α-SMA was identical in human and rat LGs, being closely associated with acini but not ducts. This indicates that potentially contractile cells were only present surrounding acini. To identify these contractile cells, we undertook double labeling of α-SMA with the mesenchymal cell marker, vimentin, and showed that there was no colocalization, in either the human or rat LGs. This result allows two deductions to be made: (1) the α-SMA-positive contractile cells were not mesenchymal in nature, and hence were likely to be myo-epithelia; (2) the vimentin-positive cells were not contractile and so were likely to be standard fibroblasts rather than myo-fibroblasts. The presence of myo-epithelial cells in acini between the basement membrane and the epithelia has been noted,^60^ although their presence adjacent to ductal epithelial cells has also been reported.^21^^,^^61^ Myo-epithelial cells carry out key roles in LG function, including mediation of signaling between local cells through the stroma and provision of structural and nutritive support for acini. Furthermore, contraction of these cells assists the release of secretory products and their propulsion into the ducts.^60^

It is important to note that we only analyzed female LG tissue in this study. Clear differences have been reported between male and female LGs in both humans and rats, as well as other species.^62^^,^^63^ These differences include an equivalent significantly elevated acinar area in the male LG in both species at all ages examined post-weaning, with a consequent greater tissue density in the female rats.^62^^,^^63^ Furthermore, it has been reported that there is an increased volume of secretory component with a greater immunoglobulin concentration from the male rat LG,^64^^,^^65^ although these findings remain unconfirmed.^63^ Development of different LG histo-architecture in the male rats is thought to be due to the influence of androgenic hormones.^64^^–^^66^ It has been shown, for example, that castration of male rats causes their LG structure to develop similarly to that of equivalent female rats and that administration of androgens such as testosterone reverses this effect.^67^^,^^68^ The sexually dimorphic nature of the LG is thought to be important, because there is a strong female species bias in incidences of both dry eye disease^18^ and Sjögren's syndrome.^69^ It has, for example, been shown that androgen treatment can counteract the effects of dry eye disease in female patients, particularly when peri-menopausal.^70^ It would thus be of interest to compare the parameters investigated in the present study between males and females in both the rat and human LG samples in a future study.

It must also be noted that the adult human female tissue samples in the present study were from donors in an age range that was statistically more likely to include menopausal subjects. Although the rat tissue samples were also from adult female rats, they did not cover the equivalent stage, because reproductive senescence occurs between 9 and 12 months of age in rodents.^71^ Although human menopause is associated with an increased likelihood of developing dry eye disease,^72^ however, there is little evidence for structural changes in the LG associated with reproductive senescence in humans,^7^ and age-related changes in rats occur most often in males.^73^ Interestingly, menopause is associated with declining levels of estrogen.^74^ Estrogen has a controversial association with dry eye disease, with some studies relating that declining levels of this hormone are associated with this condition and some stating that increased levels are responsible.^75^ This controversy has clinical implications with some studies suggesting that hormone replacement therapy aids the treatment of dry eye in peri- and post-menopausal women and some studies showing no beneficial effects.^76^ Again, it would be interesting to compare the parameters investigated in the present study in LG tissue from pre- and post-menopausal female subjects, in order to more clearly define the relationship between reproductive senescence and dry eye disease.

### Cytokeratin Expression

CKs are constituents of the epithelial cytoskeleton and help these cells to resist external stress.^77^ Expression of epithelial CKs is specific to particular organs or tissues.^78^ To compare human and rat LGs, we used a selection of well-characterized CK antibodies. Interestingly, there was a clear difference in CK expression between the two species: CK18 was the primary CK expressed by rat acinar epithelia, but human acinar epithelium also expressed CK8. Both CK18, which is a member of the type I, acidic CKs, and CK8, which is a member of the type II, neutral-basic CKs, are commonly expressed in simple (monolayer) exocrine gland epithelia along with CK7 and 19.^79^ Antibody clone MA5 recognizes all four of these isoforms, whereas antibody C-11 reacts with 8 and 18, but not 7 and 19. The fact that each antibody labeled almost identically suggests that CK7 and 19 are not highly expressed in either human or rat LGs. Of relevance, CK8 and CK18 are known to be co-expressed complementary partners.^80^ It has long been thought that in the absence of CK8, CK18 is unstable and cannot form functional cytoskeletal filaments, and vice versa^81^; however, alternative partners have now been proposed for both of these CKs.^78^ It is possible that the difference in acinar CK expression in human versus rat LG plays a role in determining the distinct physical forms of these epithelia – cuboidal/columnar in the human versus more pyramidal in the rat – but further experiments are needed to explore this possibility. CK8 and CK18 are widely expressed throughout the eye^82^ and, in agreement with our findings, CK18 expression has been detected in rat acinar cells,^83^^,^^84^ and CK7, 8, and 18 in human acinar cells.^85^

### Immune Cells in the LG

Dendritic cells are phagocytic cells involved in the activation of T cells via antigen presentation. These cells have previously been detected within glandular tissue,^86^ including LG,^87^ where they are presumed to undertake immune surveillance. Location of dendritic cells was comparable in human and rat LGs, with numerous cells positioned mainly adjacent to or between the luminal epithelia of ducts, a distribution that may also indicate past or present local infections. T cells likewise displayed a similar distribution in human and rat LGs. As with dendritic cells, they were positioned close to ducts, but, in this case, in the interstitial stroma, and are also likely undertaking immune-surveillance. A significant population of T cells has previously been reported in human LG^87^ both in lymphocytic foci and singly in the stroma. Our results confirm these findings. Lymphocytes in general have been noted in rat LGs, but to our knowledge, this is the first demonstration of the presence of T cells. It was also apparent that T cells were more numerous in the human LG. This may largely be a reflection of differing stage of life, because lymphocytic infiltration has been noted in aged rodent LGs.^88^^,^^89^

In common with T cells and dendritic cells, differentiated B cells or plasma cells were positioned adjacent to ducts in the human LG. These cells are large, antibody-secreting lymphocytes, which are well-known to be distributed in human LGs^21^^,^^87^^,^^90^^,^^91^ and, in fact, have been reported to account for over 50% of blood mononuclear cells present.^87^ We detected a much lower number of plasma cells in the rat LG, a finding in agreement with earlier work.^21^ A key function of plasma cells in the LG is the production of dimeric IgA, secreted into the tear film.^92^ The LG is, thus, a component of the mucosal-associated immune system, which acts to prevent potential pathogen entry via external mucosae such as the nasal or ocular surfaces.^93^ The relative lack of plasma cells in the rat LG, however, does question whether there is any significant IgA component in the rat tear film. In a further striking disparity, mast cells were found in the rat LG adjacent to ducts, but were absent from the human LG. Mast cells have previously been demonstrated in the rat,^58^^,^^89^^,^^94^^,^^95^ but not the human,^21^ LG.^21^^,^^22^^,^^87^^,^^96^ Mast cells play a role in many processes, such as adaptive immunity, pathogen detoxification, and allergic responses,^97^ during which they release a number of immune mediators, including a wide variety of cytokines. It is conceivable that Mast cells in rat LGs secrete tear components to moderate pathogen toxicity and entry in an analogous manner to plasma cells in the human LG.

### Nervous Innervation

Immunolocalization of the synaptic vesicle-associated glycoprotein, synaptophysin, represents a specific means to illustrate the complete nervous innervation of a tissue.^88^^,^^98^ By utilizing this technique, we showed that nervous input was present adjacent to blood vessels and to acini in human and rat LGs. Parallel localization of TH was also carried out, as this enzyme is restricted to catecholaminergic neurons and, therefore, specifically delineates sympathetic nerve input.^99^ Interestingly, sympathetic innervation of both the human and rat was essentially limited to blood vessels, with very few synapses associated with acini, as reported previously.^100^^,^^101^ The confinement of most TH-immunoreactivity to blood vessels, moreover, also defines that the direct innervation to myoepithelial cells and, therefore, acini, is largely parasympathetic, also as previously reported.^102^ It has been documented that post-ganglionic parasympathetic fibers in the LG release neurotransmitters, such as acetylcholine and vasoactive intestinal peptide, which activate receptors on both acinar and myoepithelial cells.^102^^–^^105^ Activation of neural input into acinar and myoepithelial cells stimulates secretion and contraction, respectively, thereby controlling LG function. We determined that the innervation patterns for both the human and rat LGs were very similar, meaning that essentially there would likely be negligible differences in neural stimulation of LG function when comparing the two species.

### Fibroblasts and ECM

In both human and rat LGs, fibroblasts were present in the stromal space, predominantly around ducts and, to a much lesser extent, around acini. Their presence in the LG stromal region has been well-described: the function of these cells is to provide a basic scaffold for the stroma and to secrete the majority of the constituent ECM.^106^ The labeling of fibroblasts was mirrored by the location of ECM and of collagen isotypes and laminin. We used both Masson's Trichrome histological method to stain all connective tissue, and immunohistochemical labeling to examine some of the individual components of this ECM. Our study identified laminin and collagens III to VI expression in both human and rat LGs. Although the location of fibroblast and ECM labeling was similar in rats and humans, there was one notable difference, namely a greater level of both in the human LGs. This can be explained by two of the histo-architectural findings: first, the secretory units were much more densely packed in the rat LG, meaning that there was more stromal tissue between these units in the human LGs, and, therefore, more fibroblasts and ECM; second, relatively more ducts were present in the human LGs, and the fibroblasts/ECM were more concentrated around the ducts, hence the greater amount of detectable fibroblasts/ECM as compared to the rat tissue.

### Aquaporins

AQPs constitute a family of membrane-bound channels that allow movement of water molecules into and out of cells.^107^ They are widely expressed in ocular tissues^108^^,^^109^ and in secretory glands,^46^^,^^110^ and are believed to play a major role in LG function.^111^ Previous studies have concentrated on the AQP5 isoform, which is known to be expressed in exocrine glands including the lacrimal and major salivary glands.^112^ We localized AQP5 to apical membranes of acinar epithelial cells in both human and rat LGs, in agreement with previous findings.^113^^–^^115^ However, we found a marked discrepancy in expression of other AQP isoforms between human and rat LGs. AQP1 was expressed in endothelial cells in both human and rat LGs, but was additionally present in myoepithelial cells and acinar epithelia in human. AQP1 is well-known to be expressed by endothelial cells,^116^ but is also present in myoepithelial cells in the human salivary gland.^110^ More strikingly, we found that human LGs expressed AQP3 (on acinar epithelial basolateral surfaces) but did not express any AQP4, whereas, conversely, rat LGs expressed AQP4 (in the basolateral membranes of ductal epithelia) but no AQP3. AQP4 has been identified in rodent LGs epithelial basolateral membranes in previous work,^115^ but has not been described in the human LG and, indeed, has been shown to be absent from human salivary glands,^110^ which are structurally very similar to LG. AQP3 has been detected in human LG,^108^ but there are no reports in rat LGs.

What are the roles for AQPs in the LG? Overall, the location of members of this channel family across both the human and rat LG, in endothelial cells, myoepithelial cells, and both acinar and ductal epithelia, denote that water movement in this tissue is fundamental to its function. It would be particularly logical, for example, that the expression of AQP5 on apical surfaces of acinar/ductal epithelia would define a role for this channel in aqueous tear production in both species. Intriguingly, however, knockout of AQPs 1, 3, 4, or 5 has been shown to have no effect on the volume of secreted tears,^117^ even though AQP5 knockdown in the salivary gland causes saliva to be low in volume, highly viscous, and hypertonic,^118^ and even though specific abrogation of AQP5 expression in LG has been shown to cause a disorganization of the acinar structure, an induction of endoplasmic reticulum (ER) stress and, significantly, reduce aqueous tear secretion and contribute to a dry eye disease phenotype.^111^^,^^119^^,^^120^ Furthermore, normal tear volume can be accounted for by non-AQP water flow, unlike the salivary gland which produces a much greater volume of secretome.^121^ It can be deduced that AQP5 is likely to be an osmoregulator, working to maintain the isotonicity of tears, rather than contributing to tear volume.^108^

What are the implications of differential AQP expression in human versus rat LG? Some members of the AQP family are permeable to solutes other than water. In particular, AQP4 is only permeable to water, whereas AQP3 is also permeable to small organic molecules, such as glycerol.^122^ The main roles for intracellular glycerol are as a metabolic substrate in triglyceride and phospholipid biosynthesis, gluconeogenesis, or the regeneration of NAD^+^.^123^ The data presented herein suggest that these additional metabolic targets need to be met in the human LG, hence the expression of AQP3.

### Secretory Output

Evidence of the secretory output of the LG was demonstrated by positive labeling for NKCC and mucins. NKCC was abundant on epithelial cell basolateral, but not apical, membranes in both acini and ducts in human and rat LG. The localization of NKCC in the rat LG and the human LG acinar cells is entirely consistent with previous data,^124^^–^^126^ however, to our knowledge, ours is the first confirmation of the location of NKCC expression in duct cells in the human LG. NKCC has been demonstrated to be functionally active in lacrimal fluid production,^127^ and its identical distribution pattern in human and rat LG implies that it plays the same role in both species.

The innermost layer of the tear film is composed of a mucin layer, primarily secreted by conjunctival goblet cells.^2^ However, mucins are also secreted by mucous acinar cells in the LG.^21^ Epithelial-derived mucins are generally separated into two groups: acid mucins and neutral mucins.^128^ Individual mucins can be neutral, if glycosylated with a high content of uncharged monosaccharides such as mannose, galactose or galactosamine, or acidic, if glycosylated with a high content of acidic residues such as sialic acid.^128^ Glycosylation is dependent upon the tissue and role of the individual glycoprotein.^129^ We detected neutral mucin, but not acid mucin, production in both the human and rat LGs. The tear film is known to contain high levels of acid mucins,^130^ and so our results imply that this role is fulfilled by the conjunctival goblet cells, although one study does suggest that the human LG can produce acidic mucins.^56^ We detected a species difference in cells producing mucin: in the rat, neutral mucin production could only be seen in ducts, whereas in the human, ductal and acinar epithelia stained positively. It is possible that rat acini also produce mucin, and that the observed difference was artefactual, caused by the greater density of cells preventing dye penetration. Some mucins remain bound to the cell membrane where they are synthesized,^128^ however, we found that staining for neutral mucin was present in vesicles throughout epithelial cells in both human and rat LG, although it was generally closer to the apical surface, implying it was to be secreted.

The individual mucin isotype, MUC-4, has been identified in human^59^ and rat LGs^83^ and we therefore used immunohistochemistry to analyze expression of this glycoprotein to compare its localization between the two species. In both species, MUC-4 was present in ductal epithelial cells and so could have accounted for some of the neutral mucin detected by the histological stain. Interestingly, we also detected MUC-4 to be associated with the acinar epithelial membranes in the rat but not in the human LG, suggesting an additional role for this glycoprotein in the former. MUC-4 is usually classified as a membrane-bound mucin, but in the rat LG, both membrane-bound and soluble forms of this isoform have been detected.^83^ In its membrane-bound form, this isoform has a number of biological roles which are distinct from the rest of the mucin family, including mediation of cell-cell interactions, intracellular signaling via tyrosine kinase receptor activation, and action as a growth factor reservoir.^131^ Its presence at the intercellular boundaries of rat acinar epithelia suggests that it may be involved in cell-cell interactions here, although further work is needed.

### Progenitor Cells

Progenitor cells have previously been identified in human and rodent LG.^31^^,^^33^^,^^37^^,^^49^^,^^132^^,^^133^ The presence of progenitor cells in the LG has important implications for regeneration or bio-engineering. We analyzed putative progenitor cell populations using a variety of marker proteins of varying specificities. Nestin and CD34 was clearly not indicative of progenitor cells, as both markers labeled blood endothelial cells, despite previous reports detailing these proteins as markers of progenitor cells.^49^^,^^132^^,^^133^ A sparse population of Ki67-positive cells were present in both human and rat LGs denoting potentially proliferative cells; comparison of cell numbers revealed that there was no statistically significant difference between the numbers of Ki67-positive cells in the human and rat LGs. Ki67 is expressed during active stages of the cell cycle in all vertebrate cells, and the detection of a population of cells expressing this protein in both human and rat LG suggests that either cells are actively proliferating or that they have been halted in mid-cycle. Ki67 has been used to detect stem cells,^49^^,^^133^ but again this protein is not specific to progenitor cells and further proof is required before defining Ki67-positive cells as being progenitor in nature. In our study, the Ki67-positive cells appear to be of epithelial/myoepithelial origin, due to their positioning in acini. It has previously been discussed that LG progenitor cells are predominantly of epithelial/myoepithelial origin.^49^^,^^133^^,^^134^ ABCG2 is often expressed in stem cell populations.^53^ We detected a sparse number of ABCG2-positive cells in the stroma, suggesting these cells are of fibroblast lineage. Previous work has suggested that mesenchymal stem cells exist in the LG.^132^ Importantly, the antibodies that we used to identify putative progenitor cells labeled identical cell populations in both the human and rat LGs, meaning that the regenerative potential of both would likely be comparable.

## Conclusions

In terms of general structure, the rat LG is similar to the human LG, although many of the acinar epithelial cells appear to be of a pyramidal rather than a cuboidal/columnar nature, and are therefore more densely packed, leaving a narrower central lumen. Distribution of other cell types, such as fibroblasts, myoepithelial, dendritic cells, and T-cells, is similar in both species, as are putative progenitor-type cells. Nevertheless, some clear differences between human and rat LG were apparent, for example, the distinct profiles of epithelial CKs and differences in AQP expression may be of significance. Furthermore, rat and human LGs contain different profiles of mast cells and plasma cells, which has implications for IgA secretion. Overall, the rat LG serves as a useful substitute for the human equivalent, but there exist differences which cast a cautionary light on translating results to the clinic.

## Supplementary Material

Supplement 1
