# Supplementary material for: Histochemical Comparison of Human and Rat Lacrimal Glands: Implications for Bio-Engineering Studies
Source: Transl Vis Sci Technol. 2022 Nov 14;11(11):10. doi: 10.1167/tvst.11.11.10 (PMC9669807; doi:10.1167/tvst.11.11.10)
Supplement: Supplement 1 [file tvst-11-11-10_s001.pdf]

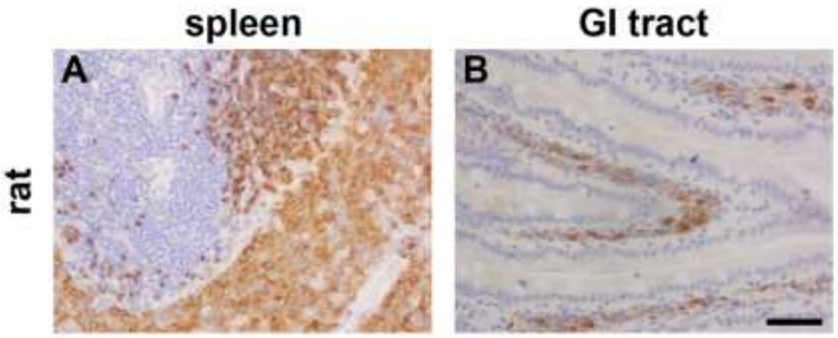

Supplemental figure 1

Figure S1. Localisation of CD79a-positive B cells in rat spleen and GI tract. CD79a-positive cells in GI tract in all likelihood represent plasma cells. Scale bar = 60µm.

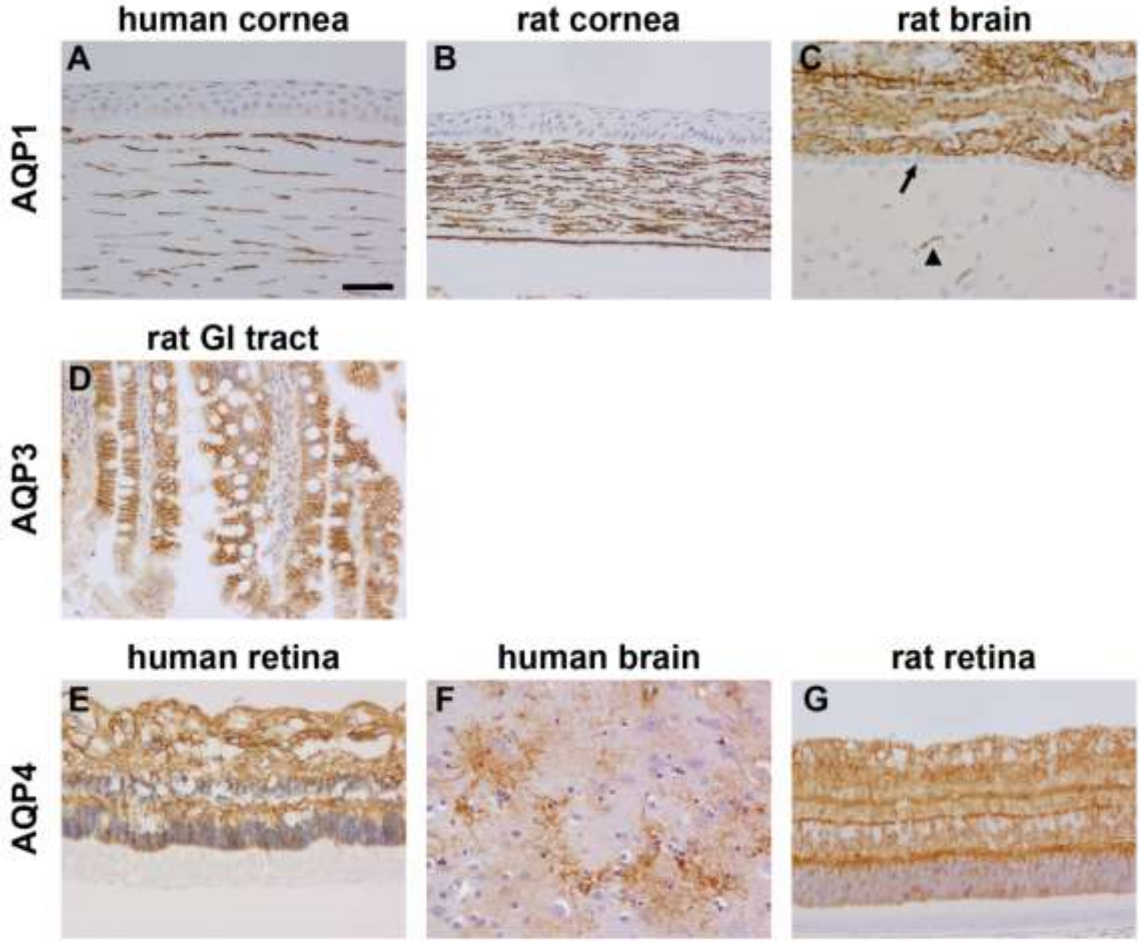

Supplemental figure 2

Figure S2. Localisation of aquaporins (AQPs) in positive control tissues. Positive labeling for AQP1 is oberved in corneal keratocytes and endothelium (A, B), as well as choroid plexus (C). AQP3 localises to the basolateral membrane of villus epithelial cells in gastro-intestinal tract (D). AQP4 is abundantly expressed in retinal Müller cells (E, G) and brain astrocytes (F). Scale bar: A, B, D-G = 60µm; C = 60µm.

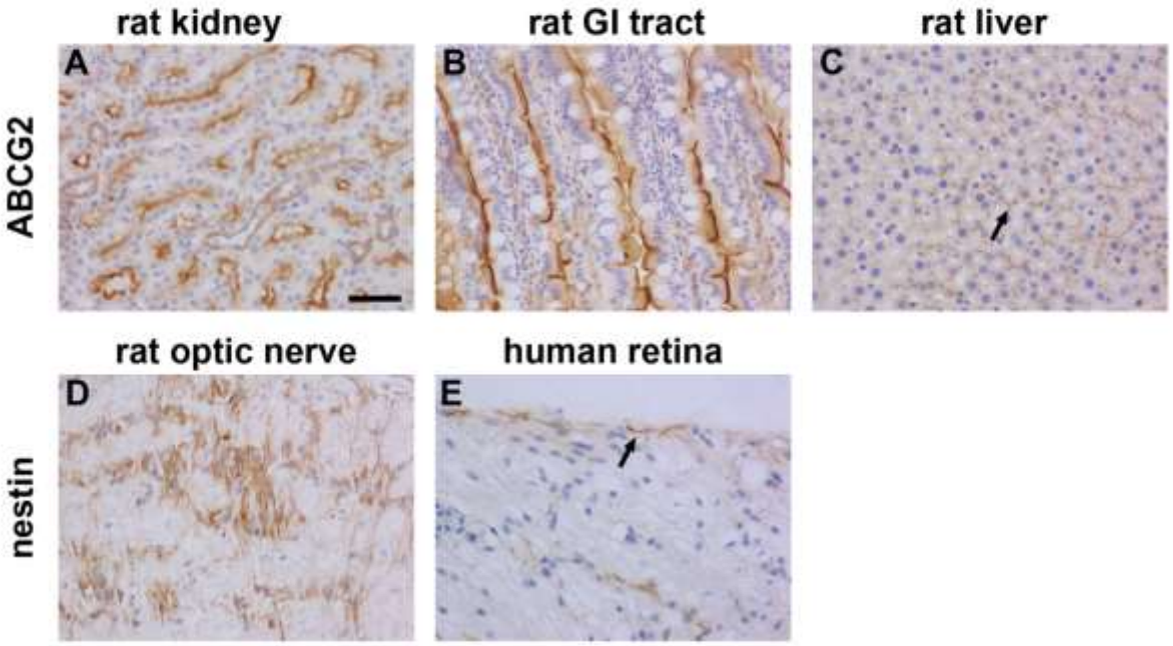

Supplemental figure 3

Figure S3. Expression of ABCG2 and nestin in positive control tissues. Localisation of ABCG2 in proximal tubules of rat kidney (A), apical membranes of epithelial cells of gastrointestinal (GI) tract (B) and hepatic bile canalicular membranes (C, arrow). Localisation of nestin in astrocytes of rat optic nerve (D) and human retina (E). Scale bar = 60µm.
